# The Feline Calicivirus Leader of the Capsid Protein Has the Functional Characteristics of a Viroporin

**DOI:** 10.3390/v14030635

**Published:** 2022-03-18

**Authors:** Yoatzin Peñaflor-Téllez, Bibiana Chávez-Munguía, Anel Lagunes-Guillén, Lizbeth Salazar-Villatoro, Ana Lorena Gutiérrez-Escolano

**Affiliations:** Departamento de Infectómica y Patogénesis Molecular, Centro de Investigación y de Estudios Avanzados del IPN, Av. IPN 2508. Col. San Pedro Zacatenco, Mexico City 07360, Mexico; yoatzin.penaflor@cinvestav.mx (Y.P.-T.); bchavez@cinvestav.mx (B.C.-M.); alagunes@cinvestav.mx (A.L.-G.); lsalazar@cinvestav.mx (L.S.-V.)

**Keywords:** feline calicivirus, LC, viroporin, disulfide bonds

## Abstract

The leader of the capsid (LC) protein is exclusive to the *Vesivirus* genus, and it is needed for successful feline calicivirus (FCV) replication, as well as an efficient apoptosis induction through the mitochondrial pathway. In this work, we aimed to determine if the LC protein from the FCV is a viroporin. Although lacking in a transmembrane domain or an amphipathic helix, the LC protein from the FCV is toxic when expressed in bacteria and it oligomerizes through disulfide bonds, which are both key characteristics of viroporins. An electron microscopy analysis of LC-expressing *E. coli* cells suggest that the protein induces osmotic stress. Moreover, we found that the previously studied C40A LC mutant, that fails to induce apoptosis and that hinders the replication cycle, also oligomerizes but it has a reduced toxicity and fails to induce osmotic stress in bacteria. We propose that the LC protein is a viroporin that acts as a disulfide bond-dependent antimicrobial peptide, similar to the Ebola virus delta peptide.

## 1. Introduction

Caliciviruses are non-enveloped, positive-sense, single-stranded RNA viruses that belong to the *Caliciviridae* family, composed of 11 genera that cause a wide range of diseases in several vertebrates [1]. Depending on the genus, genomes can contain from two and up to four open reading frames (ORF) that encode for six non-structural (NS) and two structural proteins, known as VP1 and VP2, respectively. For a successful viral replication, and depending on the genus, caliciviruses have to achieve similar cellular altered states through the actions of different viral and cellular proteins (reviewed in [2,3]). During a calicivirus infection, apoptosis is required for an efficient virus replication, either as a mechanism to evade the innate immune response [4] and/or for its dissemination into the host [5]. The mechanism by which caliciviruses induce apoptosis varies wildly between virus genera, as well as species [6].

For decades, the feline calicivirus (FCV), a member of the *Vesivirus* genus, has been one of the best go-to models to study calicivirus biology [7], as it can replicate effectively in cell cultures, and multiple reverse genetics systems have been developed [8,9,10,11]. It is known that FCV induces apoptosis in cultured cells, as the pro-apoptotic Bax protein relocates to the mitochondria during the FCV replication cycle through an unknown mechanism [12].

FCV, as with all members of the *Vesivirus* genus, encode for six non-structural proteins from its ORF1, known as NS1-NS6/7. While NS1, NS2, and NS4 have been poorly characterized, NS3 is known as the NTPase, NS5 is known as the virion protein that is genome-linked (VPg), and NS6/7 is known as the protease and the RNA-dependent RNA polymerase (protease–polymerase) (recently reviewed in [13]). The two structural proteins, VP1 and VP2, are encoded from the ORF2 and ORF3, respectively. VP1, the major capsid protein, is expressed as a precursor protein from the subgenomic RNA at a late stage post-infection [14], and is immediately processed by the viral protease–polymerase NS6/7 [15] in the mature VP1 protein and in the leader of the capsid (LC) protein, which is a peptide of 124 amino acids from the N terminal region of 14.7 kDa, responsible for the cytopathic effect, as well as apoptosis induction [16,17]. The LC protein from FCV has no sequence homology to the other proteins reported in known databases; however, it contains two conserved regions among the *Vesiviruses.* In the LC protein from the FCV Urbana strain, the first of two conserved regions (CRI) spans the amino acid residues 33–40 containing conserved cysteines at positions 33, 39, and 40. The second conserved region (CRII) consists of proline residues in positions 108, 109, and 112, with nonpolar amino acids at positions 110 and 111 [16]. Point mutations in any of the conserved cysteine residues in the FCV LC protein resulted in a reduced replication, marked by a failure of the cytopathic effect establishment and a reduction in the virus yield. Abente et al. [16] reported that the mutant LC-C40A virus, containing a cysteine 40 per alanine change, can recover a WT phenotype through several passages with two point mutations that resulted in the replacement of a serine by a proline at position 29, as well as gaining a new cysteine residue at position 41 from a threonine replacement [16]. The positive selection of the cysteine residues in the CRI suggest that these amino acids are necessary for the protein’s function and the overall successful virus replication [16].

More recently, our working group demonstrated that the expression of the WT-LC protein from FCV (the Urbana strain), induced a cell rounding phenotype that resembled the cytopathic effect observed during an FCV infection. Transmission electron microscopy (TEM), coupled to immunogold labelling, showed that WT-LC is located at the periphery of reduced electron density mitochondria and can present an altered morphology, similar to that observed in infected cells at late-stage post-infection. Moreover, cell fractionation and a Western blot analysis showed that WT-LC expression induce apoptosis through the intrinsic pathway, as previously reported, since the proapoptotic protein Smac/DIABLO relocates from the mitochondria to the cytoplasm of the WT-LC-expressing cells, leading to the inhibition of apoptosis protein (IAP) degradation, the effector caspase 3 activation, and PARP cleavage [17]. Interestingly, the LC mutant, C40A (LC-C40A), when expressed in cells, caused the cell rounding phenotype, although to a lesser extent than that observed in the WT-LC-expressing cells. However, it failed to induce apoptosis, suggesting that the LC protein might have more than one role in the FCV replication cycle [17]. The ability of the WT-LC protein, from the FCV, to induce the cell rounding phenotype, apoptosis and its localization in the mitochondria, as well as the importance of the cysteine residues in its CRI, similar to the conserved cysteines in the recently described Ebola virus (EBOV) delta peptide viroporin activity [18] suggest that this protein is a viroporin.

Viroporins are viral proteins that share common biochemical and functional characteristics, forming pores or channels in the cell membranes that are crucial for viral replication. Viroporin classification was originally stated by the number (one or two) of transmembrane domains (TMDs) in its sequence [19,20,21] and other examples previously reviewed in [22]. However, the latest characterization of novel viroporins have demonstrated that they can have more, such as the NS2A protein from the dengue virus, that contains seven predicted TMDs [23]. On the other hand, the study of the EBOV delta peptide’s viroporin activity showed that its ability to permeate membranes does not depend on its TMD, but on its intramolecular disulfide bond that interacts with membrane phospholipids, which occurs with the antimicrobial peptides [18].

In this study, we performed an in silico analysis, as well as in vitro experiments, to determine if the LC protein from FCV has the structural and functional characteristics of a viroporin. We found that although the LC protein from the FCV lacks the biochemical characteristics of a viroporin, such as a TMD or an amphipathic helix, as well as a polybasic region distributed in a one-sided alfa helix, it contains two important viroporin properties, such as toxicity and oligomerization.

Our results suggest that the LC protein from the FCV acts as a viroporin that does not depend on TMDs, an amphipathic helix, or polybasic regions, but rather on disulfide bonds, to induce mitochondria disruption and apoptosis, as does the delta peptide from EBOV. Thus, we propose that LC is a “non-classical” viroporin.

## 2. Materials and Methods

### 2.1. In Silico Analysis

The prediction of the TMDs was made with TMHMM 2.0 from ExPASy (http://www.cbs.dtu.dk/services/TMHMM/ accessed on 3 April 2021) and the distribution of basic amino acids in a helical wheel arrangement was made with the NetWheels software (http://lbqp.unb.br/NetWheels/ accessed on 3 April 2021). 

The disulfide bond prediction was made with DISULFIND ([24] http://disulfind.dsi.unifi.it accessed on 4 April 2021). The secondary structure prediction was made with Biogem CFSSP: Chou & Fasman Secondary Structure Prediction Server ([25] http://www.biogem.org/tool/chou-fasman/ accessed on 21 March 2021), and the amphipathic helix prediction was made with AmphipaSeek (https://npsa-prabi.ibcp.fr/cgi-bin/npsa_automat.pl?page=/NPSA/npsa_amphipaseek.html accessed on 3 April 2021). The de novo structure of WT-LC and the mutant LC-C40A CRI regions were made with PepFold 3.5 [26] (https://mobyle.rpbs.univ-paris-diderot.fr/cgi-bin/portal.py#forms::PEP-FOLD3 accessed on 21 June 2021), respectively.

Zaire EBOV delta peptide and LC sequences were retrieved from the National Center for Biotechnology Information database (NCBI, National Institute of Health, Bethesda, Maryland, United States of America) and was aligned with T-Coffee [27]. Results were visualized with JalView [28].

### 2.2. Cells and Cultures

The Crandell and Reese Feline Kidney (CrFK) cells (American Type Culture Collection (ATCC) (Rockville, MD) were cultured in six-well plates using Eagle’s minimal essential medium MEM Thermo Fisher Scientific (Waltham, MA, USA), supplemented with Earle’s balanced salt solution, 0.1 mM of nonessential amino acids, 2 mM of L-glutamine, 1.0 mM of sodium pyruvate, 1.5 g/L of sodium bicarbonate, and 10% (*v/v*) of Gibco Fetal Bovine Serum (FBS), as well as 5,000 units of penicillin and 5 μg/mL of the streptomycin antibiotic at 37 °C and at 5% CO_2_.

All of the *E. coli* Bl21(DE3)pLysS assays were performed in a Luria–Bertani (LB) liquid medium (1% tryptone *w*/*v*, 1% NaCl *w*/*v*, and 0.5% yeast extract *w*/*v*) without or with ampicillin (50 mg/mL) as indicated, and was incubated at 200 RPM at 37 °C; or, in LB–agar plates (1% tryptone *w*/*v*, 1% NaCl *w*/*v*, 0.5% yeast extract *w*/*v*, 1.5% agar *w*/*v*, and ampicillin 50 mg/mL) without or with 1 mM Isopropyl-β-D-thiogalactopyranoside (IPTG) (Thermo Fisher Scientific, MA, USA) as indicated, and was incubated at 37 °C.

### 2.3. Plasmid Constructions and Purification

To obtain pRSETA-LC and pRSETA-LC-C40A prokaryotic expression plasmids, WT-LC and mutant-LC-C40A codifying sequences were obtained from Wt-LC-pAm-Cyan and Mut-LC-pAm-Cyan vectors previously, respectively [17], through plasmid digestion with XhoI (Jena Bioscience, Jena, Germany ) and HindIII (New England Biolabs, Ipswich, MA, USA) restriction enzymes using the Jenas Bioscience Universal Buffer™ and were purified from agarose gels with the QIAGEN (Hilden, Germany) purification kit, following the manufacturer’s instructions. A pRSETA prokaryotic expression vector was also digested with XhoI and HindIII restriction enzymes and was purified, as described above. DNA fragments were quantified by spectrophotometry, and ligation reactions were made with the T4 ligase (Thermo Fisher Scientific, MA, USA) following the manufacturer’s instructions. Ligation reaction products were used to transform chemocompetent *E. coli* DH5-α cells by heat shock at 42 °C for 60 s and plated in ampicillin 50 mg/mL LB–agar plates. Selected colonies were grown in a LB medium, supplemented with ampicillin, overnight at 37 °C. Plasmid extraction was performed by alkaline lysis [29]. The extracted plasmid was digested by XhoI and HindIII restriction enzymes to determine the presence of the WT-LC and mutant LC-C40A CDS inserts, and their sequences were confirmed by the Sequencing and Synthesis Unit of the Instituto de Biotecnología de la Universidad Nacional Autónoma de México, using the T7 promoter. The results were visualized with FinchTV (Geospiza Inc., Seattle, WA, USA).

To purify the eukaryotic expression vectors pAM-Cyan, Wt-LC-pAm-Cyan, and Mut-LC-pAm-Cyan for CrFK cell transfection, 200 mL of an LB + Amp medium, from the previously transformed *E. coli* DH5-α strain, were incubated for 16 h at 200 RPM and at 37 °C. Cultures were used for plasmid purification using the Jena Bioscience Midi Prep Extraction kit, following the manufacturer’s instructions.

### 2.4. Protein Expression and Purification

Chemocompetent *E. coli* Bl-21(DE3)pLysS cells were transformed by heat shock and were plated in LB–agar–ampicillin plates, as described above. Grown colonies were inoculated in 5 mL of an LB + Amp medium and were incubated overnight at 200 RPM at 37 °C. The culture was diluted to 1:10 in a fresh LB + Amp medium, and the protein expression was induced by adding 100 mM of Isopropyl β-D-1-thiogalactopyranoside (IPTG) to a final concentration of 1 mM, and was incubated at 200 RPM for 4 h at 37 °C. Cells were pelleted, washed with phosphate buffer saline (PBS) solution (137 mM NaCl, 2.7 mM KCl, 10 mM Na2 HPO4, and 1.8 mM KH2 PO4), resuspended in a Laemmli lysis buffer 4X (125 mM Tris-HCl pH 6.8, 20% *v/v* glycerol, 10% *v/v* β-mercaptoethanol (βME), 4% SDS *w*/*v*, and 0.004% *w*/*v* Bromophenol blue) and was heated at 95 °C for 10 min before SDS-PAGE or storage at −20 °C. The remaining cell culture was used for 15% *v/v* glycerol/LB + Ampicilin stocks stored at −80 °C.

For protein purification, 100 mL of *E. coli* Bl21(DE3)pLysS cultures were submitted to SDS-PAGE in a 10 lane 12% acrylamide gel. The first lane was stained with Coomassie blue (40% *v/v* MeOh, 10% *v/v* Acetic acid, and 0.25% Coomassie Brilliant Blue R) for 10 min, and was then washed with distain solution (40% *v/v* MeOh and 10% *v/v* Acetic acid) until the protein bands where visible. The lane was incubated in ddH_2_O for 10 min at room temperature and was aligned with the remaining acrylamide gel to excise the unstained Histag-LC or Histag-LC-C40A bands with a sterile scalpel, which were then chopped into cubic pieces of approximately 1 mm. The protein was extracted from the polyacrylamide gels by electroelution using the Little Blue Tank (ISCO, Lincoln, NE, USA) at 90 V for 70 min at room temperature using a running buffer (Glycine 0.192 M, Tris Base 0.025 M, and SDS *w*/*v* 1%) and Spectra/Pore MWCO 12,000–41,000 Da membranes. The electroeluted proteins were quantified by using the PIERCE BSA Protein Assay Kit (Thermo Fisher Scientific, MA, USA) and SDS-PAGE was performed with both Coomassie blue staining and the Western blot analysis to assess the migration and to detect the purified protein.

### 2.5. Eukaryotic Cell Transient Transfection

CrFK cells were grown to a 70% confluence in six-well plates and were transfected for 24 h with 3.5 µg of the expression plasmids pAm-Cyan, Wt-LC-pAm-Cyan, or Mut-LC-pAm-Cyan, using the Lipofectamine2000 Reagent (Thermo Fisher Scientific MA, USA), following the manufacturer’s instructions.

### 2.6. Histag-LC Oligomer Immunodetection by Western Blotting

Two, four, and six µg of purified Histag-LC and Histag-LC-C40A proteins were resuspended in a Laemmli lysis buffer without βME, submitted to SDS-PAGE, and transferred to a 0.22µm-pored nitrocellulose membrane. Membranes were blocked with 5% skimmed milk in TBS-Tween for 30 min and were incubated for 24 h at 4 °C with an anti-His probe, antibody H-3 (Santa Cruz Biotechnology, Santa Cruz, CA, USA), diluted to 1:1000 in TBS 0.05% Tween20 (*v/v*) dilution. The blots were washed three times with TBS-Tween20 and were incubated overnight at 4 °C with the PIERCE anti-mouse IgG HRP conjugated antibody diluted to 1:10,000 in TBS-Tween 1% skim milk. Membrane development was performed using the SuperSignal™ West Femto Maximum Sensitivity Substrate (Thermo Fisher Scientific, MA, USA) and Carestream (New York, NY, USA) Kodak light sensible films. 

### 2.7. E. coli Assays

pRSETA-, pRSETA-LC-, or pRSETA-LC-C40A-transformed *E. coli* Bl21(DE3)pLysS cells were grown overnight in LB–ampicillin at 37 °C at 200 RPM and were diluted to 1:200 in a fresh LB–ampicillin medium at room temperature in 96 well plates. OD_600_ was measured by the BioRad iMark™ (Hercules, CA, USA) Microplate Absorbance Reader until an OD_600_ of 0.4 was reached, and the non-inoculated LB–ampicillin was considered as blank. The protein expression was induced by 1 mM of IPTG at the final concentration and OD_600_ readings were made every 10 min for 120 min. OD_600_ measurements were plotted over time, comparing induced with not-induced bacteria, and statistical analyses was made with GraphPad Prism 7.0 software. 

For the colony-forming unit quantification, transformed E. coli Bl21(DE3)pLysS cells were grown overnight, as described above, and were serially diluted in an LB medium until reaching 2 × 10^−6^ factor. LB–ampicillin–agar plates, without or with 1 mM of IPTG, were inoculated with previously diluted bacteria and were incubated overnight at 37 °C. Colonies were counted using ImageJ software (http:/rsb.info.nih.gov/ij, 26 December 2021, National Institute of Health, Bethesda, MA, USA) and the percentage of the ratio between plates with or without 1 mM of IPTG for each clone was calculated. A student’s *t*-test was performed between each condition using the GraphPad Prism 7.0 software.

For the osmotic shock assays of the *E. coli* Bl21(DE3)pLysS strain, 20 h cultures in LB where centrifuged at 5000 RPM for 5 min, then were resuspended in 1X PBS, centrifuged as previously stated, and were resuspended in ddH2O /Glycerol: 1:0.94, 1:1.23, and 1:1.34 for 30 min. Cells were pelleted, as before, and they immediately resuspended in glutaraldehyde to fix them for the Transmission Electron Microscopy (TEM) analysis.

### 2.8. Transmission Electron Microscopy and Immunogold Labeling

Non-transformed, osmotically-shocked, or transformed *E. coli* Bl21(DE3)pLysS bacteria with pRSETA, pRSETA-LC, or pRSETA-LC-C40A were pelleted at 4 h post-induction and were washed with 1X PBS, as described previously. Cells were pelleted and resuspended with paraformaldehyde (PFA) at 4% and glutaraldehyde at 0.5% in PBS for 1 h at room temperature and overnight at 4 °C. They were then dehydrated with ethanol in increasing concentrations and were embedded in white resin (London Resin Co., Stansted, UK) and were polymerized with U.V. light for 48 h at 4 °C. Approximately 60-nm slices were obtained from each sample and were incubated with an undiluted anti-His probe antibody H-3 (Santa Cruz Biotechnology, Santa Cruz, CA, USA) for 16 h. 

CrFK cells grown at 70% confluence were transfected with either pAm-Cyan, Wt-LC-pAm-Cyan, or Mut-LC-pAm-Cyan eukaryotic basal expression plasmids with a Lipofectamine 2000 reagent (Themo Fisher Scientific, MA, USA) following the manufacturer’s instructions. Forty-eight hours post-transfection, cells were fixed with glutaraldehyde and were processed as described previously for the TEM analysis and for immunogold labelling. 

After antibody incubation, all samples were contrasted with lead citrate and uranyl-acetate and were analyzed in a Joel JEM-1011 transmission electron microscope.

## 3. Results

### 3.1. LC from FCV Does Not Have Common Biochemical Viroporin Characteristics but Contains the γ-Core Signature of Defensins

**Figure 1:**
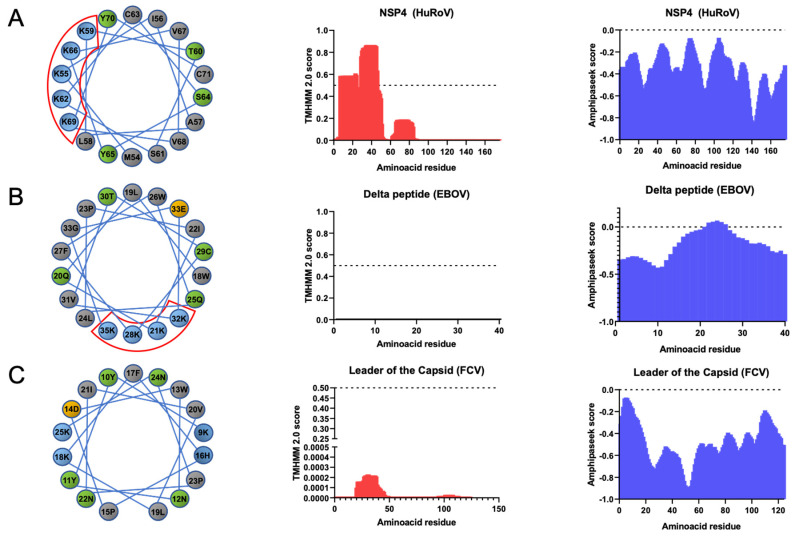
LC protein from FCV does not have the biochemical characteristics of several viroporins. Bioinformatical analysis of the NSP4 protein from (**A**) HuRoV, (**B**) delta peptide from Zaire EBOV, or (**C**) the WT-LC protein from FCV Urbana strain. Distribution of amino acids in a helical wheel using NetWheels (left), prediction of TMDs with TMHMM 2.0 with score threshold of 0.5 (center), and prediction for amphipathic helixes with AmphipaSeeK with a score threshold of 0 (right) are shown. Polybasic region of NSP4 and delta peptide enclosed in red.

The presence of transmembrane domains (TMDs), or amphipathic helixes, as well as a helical wheel of a one-sided distribution of basic amino acids in viral proteins, have been associated with viroporin function, as in the delta peptide from EBOV, or in NSP4 of the human rotavirus (HuRoV) (Figure 1A,B) [30]. Therefore, to determine if the LC protein from the FCV contains these features, various in silico analyses were performed. The LC protein from the FCV contains a region of four basic amino acids at 10, 17, 19, and 26 residues; however, they are not distributed in one side of a putative helical wheel (Figure 1C, left). Moreover, the predictors of TMDs (Figure 1C, center) and the amphipathic helixes (Figure 1C, right) suggest that the LC protein from the FCV does not have the characteristic TMDs commonly present in viroporins (Figure 1, center) or any amphipathic helixes.

### 3.2. LC from FCV Has the Molecular Signature of Defensins Similar to the Delta Peptide from EBOV

**Figure 2:**
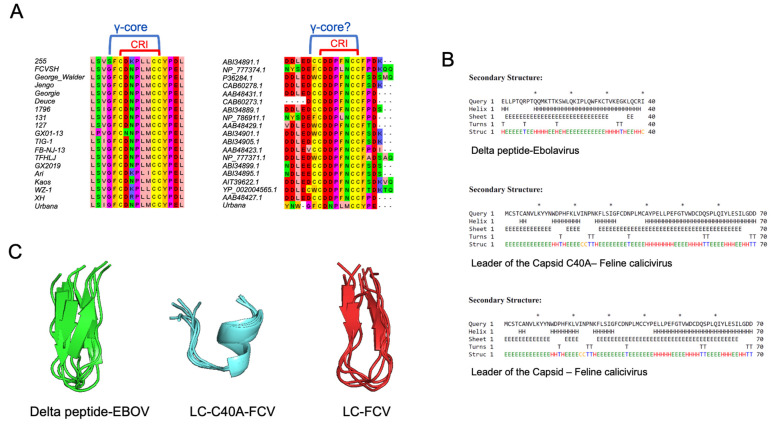
LC from FCV has defensin-conserved multidimensional γ-core motif. (**A**) Representative image of the alignment of the CRI motif sequences of LC protein from diverse FCV strains and isolates (left) or from other vesiviruses (right). (**B**) Secondary structure prediction of the delta peptide from EBOV and the mutant LC-C40A and WT-LC proteins from FCV (upper, middle, and lower panels, respectively). “H” indicates alpha helixes, “E” beta sheets, “C” coils, and “T” turns. (**C**) Tertiary structure prediction of the cysteine-containing conserved region from delta peptide from EBOV (green), the CRI from WT-LC (red), and mutant-LC-C40A (blue) proteins from FCV by the use of PEPFOLD 3.5 software. The five proposed models were modelled and aligned with Pymol (DeLano Scientific LLC, San Francisco, CA, USA).

The delta peptide from EBOV is considered an unusual viroporin; although it contains an amphipathic helix, functional assays suggest that it is not essential for its membrane permeabilization capability. Rather, it mediates its activity through disulfide bonds, similar to antimicrobial peptides such as defensins [18]. Defensins are a vast group of antimicrobial peptides that have a sequential and structural molecular signature called the γ-core motif, consisting of a GXCX_3-9_C sequence distributed in two antiparallel beta sheets necessary for its antimicrobial function. Thus, to determine if the LC proteins from the different FCV strains and isolates contain this γ-core motif, an alignment of several LC protein sequences was performed. The protein alignment of the complete LC sequences from *Vesivirus* and the FCV showed identity levels similar to the levels reported by Abente et al. [16] (data not shown), with the two conserved regions of LC present among the first 100 BLAST-aligned *Vesivirus* sequences reported in the NCBI database and with little-to-no variation among the LC sequence from 61 FCV strains or isolates. We found that the γ-core motif of defensins is present in the CRI of almost all the FCV strains and isolates analyzed (Figure 2A), except for the FCV 255/27-42 strain, which does not contain the first glycine residue. The absence of this glycine residue was also observed in the CRI region from the LC proteins of other vesiviruses (Figure 2A). In this regard, it is known that the delta peptide from EBOV also lacks this glycine residue (Figure 1, [18]); therefore, this amino acid may not be essential for the antimicrobial peptide-like function of vesivirus LC proteins.

Once the evidence that the FCV LC protein contains, in its CRI, the γ-core motif sequence of the defensins was corroborated, secondary and de novo tertiary structures from the FCV WT-LC were predicted. The secondary structure prediction of the FCV WT-LC protein suggests that its CRI is located between two beta sheets (Figure 2B). The predicted tertiary structure of the CRI region and the EBOV delta peptide conserved cysteine region, using PepFold 3.5, showed similar γ-core motif structures (Figure 2C) that have been previously reported in other defensins and sulfide bond-stabilized antimicrobial peptides. Interestingly, this structural motif is not formed in the mutant LC-C40A CRI prediction that contains an alpha helix in the CRI region (Figure 2C), correlating with its reported lack of cytopathic effect inductions, its successful viral replication, and its failure to induce apoptosis [16,17] ). Due to the poor homology of the LC to other known proteins, the full protein tertiary structure prediction of both the WT-LC and the mutant LC-C40A from the FCV had very low scores through various servers, and thus, it resulted in unreliable data (data not shown). However, the CRI-predicted structure showed a similar distribution to the PEPFOLD modelling. 

Defensins are small cysteine-rich cationic proteins that are highly distributed in a variety of lifeforms; they have different domains and motifs for membrane insertion and permeabilization. A brief bioinformatical analysis performed in distinct defensin proteins has shown that they have heterogeneous features; they can present a TMD, an amphipathic helix, both, or neither. Particularly, alpha defensins such as the *Homo sapiens* neutrophil alpha defensin 3, *Homo sapiens* neutrophil defensin 4, and the *Gallus gallus* gallinacin-3 (Figure S1, Supplementary Table S1) do not appear to contain these features. Thus, the WT-LC protein from the FCV may act as an alpha defensin.

Taken together, these results suggest that the LC protein from the FCV does not have the most common biochemical properties of viroporins. Instead, it has the γ-core antimicrobial peptide multidimensional signature. 

### 3.3. WT-LC and the Mutant LC-C40A from FCV Oligomerizes Differentially through Disulfide Bonds

**Figure 3:**
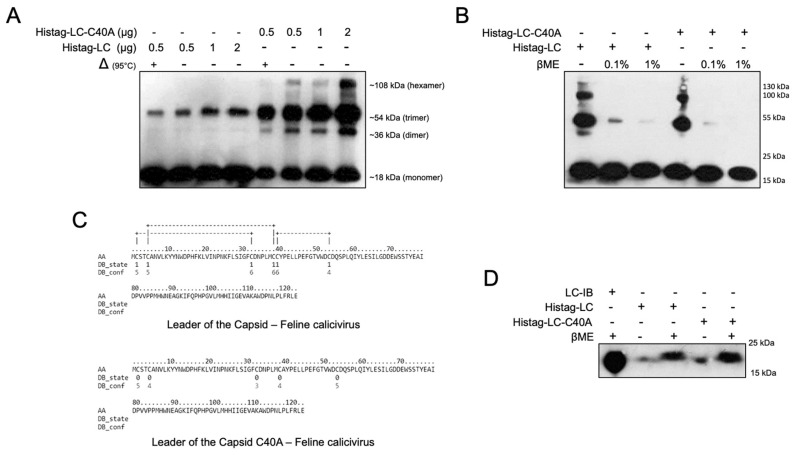
WT-LC and mutant LC-C40A from FCV homo-oligomerizes through disulfide bonds. (**A**) 0.5, 1, or 2 µg of purified Histag-LC or Histag-LC-C40A proteins were incubated 10 min at 95 °C (Δ) (+) or at room temperature (−) and were subjected to non-reducing SDS-PAGE and Western blot analysis using anti-Histag antibodies. Homo-oligomer formation is indicated. (**B**) Purified Histag-LC (1µg) or Histag-LC-C40A (0.5 µg) proteins were incubated with increasing concentrations of βME (0.1% and 1%) for 5 min and subjected to SDS-PAGE and Western blot analysis using anti-Histag antibody. (**C**) Disulfide bond prediction of WT-LC and mutant LC-C40A proteins from FCV with a DISULFIND server with state (0 or 1) and confidence (1–10) values. (**D**) Purified Histag-LC or Histag-LC-C40A proteins were incubated with or without 1% βME for 5 min and analyzed by SDS-PAGE and Western blot using anti-Histag antibody.

A defining characteristic of any given protein-forming pores or ion channels is its capacity to homo-oligomerize. Thus, the in vitro capacity of the FCV LC to homo-oligomerize was evaluated in vitro. The coding sequences from both the WT-LC and the mutant-LC-C40A were cloned in the prokaryotic inducible expression vector, pRSETA, that adds a histidine tag in the N-terminus of the protein. At 3 h post-induction (h.p.i.) with 1mM of IPTG, the levels of both Histag-LC and Histag-LC-C40A peaked, and bacterial fractionation showed that most of these proteins were located in the inclusion bodies (data not shown), as reported for several heterologous proteins expressed in *E. coli* cells. Histag-LC and Histag-LC-C40A proteins were purified through electroelution and their capacities to form homo-oligomers were evaluated by a WB assay in the presence or absence of the reducing agent, βME. We found that both Histag-LC and Histag-LC-C40A formed heat-resistant oligomers, which is the trimeric form which is the most abundant in both cases. Dimeric, pentameric, and hexameric oligomers were also identified (Figure 3A). A more evident signal in all oligomeric forms of the Histag-LC-C40A protein was detected (Figure 3A). A prediction analysis, using DISULFIND software, showed that while the Histag-LC protein could form three intramolecular disulfide bonds, the Histag-LC-C40A mutant did not present any intramolecular disulfide bond predictions (Figure 3C). Regardless, the oligomer formation of both the WT and the mutant-C40A Histag-LC proteins were depleted in the presence of the reducing agent βME (Figure 3B), indicating that this oligomerization was due to disulfide bond formation. To determine the intramolecular disulfide bond formation in both Histag-LC and Histag-LC-C40A proteins, a WB assay, with or without βME, was performed to assess monomer migration shifts. A slight change in migration between the reduced and nonreduced forms was observed with both WT and mutant Histag-LC protein monomers (Figure 3D), suggesting the presence of intra-molecular disulfide bonds in both proteins.

Taken together, these results suggest that both the WT-LC and mutant-LC-C40A from the FCV forms inter- and intramolecular disulfide bonds that participate in a homo-oligomer formation, a main characteristic of all channels and viroporins. Moreover, the presence of intramolecular disulfide bonds is an essential characteristic in antimicrobial peptides, such as defensins and the delta peptides from the EBOV viroporins. 

### 3.4. LC from FCV Is Intrinsically Toxic, Inducing a Phenotype That Resembles Osmotic Stress in Bacteria and Mitochondria

**Figure 4:**
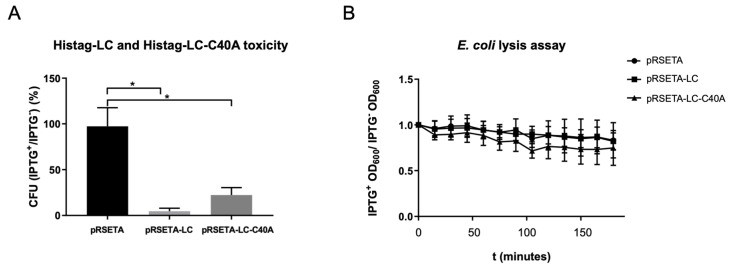
WT-LC and mutant LC-C40A from FCV are intrinsically toxic. (**A**) Overnight cultures of transformed *E. coli* bacteria with pRSET-A, pRSET-A-LC, or pRSET-A-C40A plasmids were serially diluted until they reached 1:2 × 10^−6^ and were plated in LB + agar with or without IPTG. Colonies for each plate were counted and ratios between colonies with and without IPTG were calculated for each transformed bacteria condition. Standard deviations were obtained from duplicates of at least three independent assays. Values of *p* ≤ 0.005 (*) of multiple student’s *t*-tests were calculated using GraphPad Prism 7. (**B**) Changes in the OD_600_ after Histag-LC or Histag-LC-C40A induction in *E. coli* bacteria. Transformed *E. coli* cells were grown in 96-well plates and half of them were induced with IPTG 1 mM when they reached a 0.4 OD_600_. Plate readings of OD_600_ were done every 15 min for a total of 3 h and a comparison of the induced versus non-induced bacteria and statistical analysis was done with Graphpad Prism 7, with no statistical significance found for the two-way ANOVA test.

Another characteristic reported for several viroporins is its intrinsic toxicity, or its ability to reduce cell viability in organisms that do not express this protein under normal conditions; thus, a CFU count assay was performed. The WT-LC protein caused a 95% reduction in *E. coli* CFU, while the mutant-C40A-LC protein caused only a 40% (Figure 4A), indicating that even though both proteins are toxic when expressed in bacteria, WT-LC has a greater toxicity.

To identify if the LC from the FCV protein has the ability of membrane permeation, we performed a DO_600_ assay in a viroporin-expressing *E. coli* BL21(DE3)pLysS strain, commonly used in viroporin studies (Figure 4B), based on the principle of viroporin pore formation that facilitates the relocation of the T7 lysozyme expressed in *E. coli* from the cytoplasm to the extracellular medium, resulting in bacteria lysis and OD_600_ reduction. Bacteria was grown in 96-well plates up to 0.4 OD_600_ and was induced with IPTG. OD was measured through time and, unexpectedly, we did not find differences between the OD_600_ in bacteria transformed with pRSETA-LC or PRSETA-LC-C40A when compared to the pRSETA (control) plasmids through the first 120 min post-IPTG induction. These results suggest that the LC protein from the FCV does not have the capacity to produce a pore with an exclusion size of 27kDa that facilitates T7 lysozyme relocation. However, more studies are needed to assess the LC permeabilization capability.

**Figure 5:**
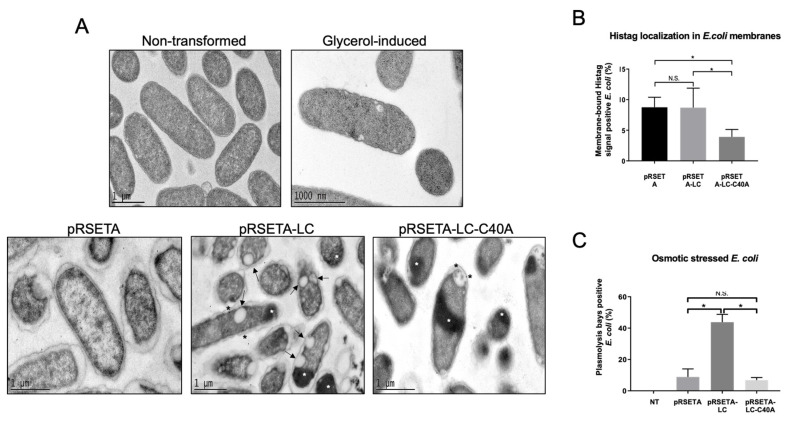
LC from FCV induces osmotic stress-like phenotype in *E. coli.* (**A**) TEM and immunogold labelling assays of pRSETA, pRSETA-LC, and pRSETA-LC-C40 transformed *E. coli* at 4 h post-induction with 1 mM IPTG using anti-Histag antibody. Non-transformed and glycerol osmotic stress-induced bacteria were used as controls. Inclusion bodies (white asterisks), nanogold particles (black asterisks), and plasmolytic bays (arrows) are indicated. (**B**) Statistical analysis of TEM micrographs to determine *E. coli* cells with Histag signal in their membranes, or (**C**) plasmolysis bays were quantified in each condition. Standard deviations were obtained from duplicates of at least three independent assays. Values of *p* ≤ 0.05 (*) or *p* < 0.0001 (*) calculated using GraphPad Prism 7.

To determine the type of damage that the LC protein from the FCV causes in bacteria, and to define if these proteins were located in their membranes, a TEM coupled with an immunogold labelling assay was performed in bacteria expressing both WT-LC and mutant-LC-C40A from the FCV, as well as the empty Histag epitope of transformed bacteria with the pRSETA vector (Figure 5A). As was previously observed by cell fractionation, we found that both WT-LC and mutant-LC-C40A proteins from the FCV are encapsulated in inclusion bodies. However, while the inclusion bodies formed with the WT-LC protein-transformed bacteria were observed mostly in one end, in the mutant LC-C40A protein-transformed cells were observed mostly in the mid body of the bacteria (Figure 5A, white asterisks).

The immunolabeling of Histag showed that both the WT-LC and mutant-LC-C40A were located in the bacterial inner and outer membranes (Figure 5A, black asterisks). Although the differences in the signals were not statistically significant compared to the empty pRSETA vector-transformed cells alone (Figure 5B left panel), it was evident that Histag detection in membranes was significantly lower in the mutant LC-expressing bacteria.

Interestingly, cells expressing the WT-LC, but not the mutant-LC-C40 form, of the FCV protein showed invaginations of its periplasmic space deriving in cytoplasmic vacuole-like bodies (Figure 5A, arrows). These structures are similar to the plasmolysis bays described in bacteria under mild-to-medium osmotic stress conditions [31]. A similar response was observed when osmotic shock was induced in non-transformed *E. coli* Bl21 cells with distinct glycerol percentages, as previously reported ([32], data not shown), suggesting that the WT-LC, but not the mutant-LC-C40A, from the FCV induces osmotic stress in *E. coli* cells. Taken together, these results demonstrate that the LC protein from the FCV is intrinsically toxic, which is another functional characteristic of viroporins, therefore inducing osmotic stress when expressed in bacteria.

### 3.5. Mitochondria of LC-Expressing CrFK Cells Undergo Apparent Mitochondrial Permeation Transition

**Figure 6:**
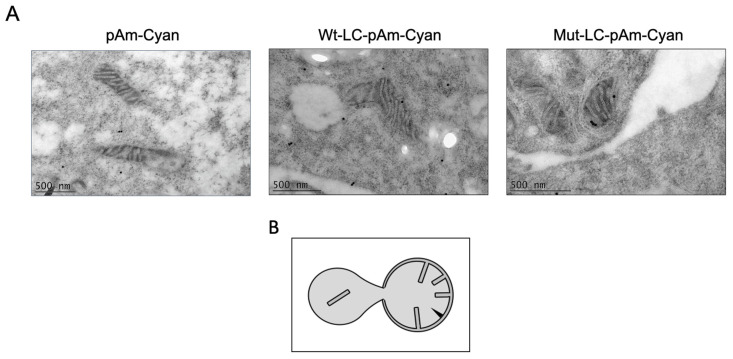
WT-LC, but not mutant-LC-C40A proteins, could induce internal membrane herniation in mitochondria. (**A**) Transmission electron microscopy images of the mitochondria integrity from CrFK cells transfected with pAm-Cyan (upper panel), Wt-LC-pAm-Cyan (center), or Mut-LC-pAm-Cyan (lower panel). After 24 h of transfection, cells were fixed and processed for TEM and immunogold labelling. (**B**) Schematic of apoptotic mitochondrial profile first reported in [33].

As described before, WT-LC proteins are located in the mitochondria from CrFK-transfected cells, and they cause the relocation of mitochondrial periplasmic proteins to the cytoplasm [17]. Therefore, we examined, by TEM coupled with immunogold labeling assays, the subcellular localization, as well as the mitochondrial morphology of Cyan, Cyan-LC, and Cyan-LC-C40A proteins in CrFK transfected cells (Figure 6). The WT-LC-pAm-Cyan protein expressed in CrFK cells was located in the outer and inner membranes, as previously described by our workgroup. Moreover, mitochondrial internal membrane herniation is observed (Figure 6), suggesting that cells are undergoing a mitochondrial permeation transition (MPT), which is a loss of mitochondrial internal membrane selective permeation capabilities, as observed in FCV-infected cells [17], and in cells undergoing apoptosis [33]. We also found that even though the mutant-LC-C40A is also located in the mitochondrial membranes, there was no appreciable MPT morphology. These results suggest that the C40A mutation in the LC from the FCV protein, that lacks the ability of apoptosis induction and an efficient viral replication, does not alter LC localization, but its function.

## 4. Discussion

All caliciviruses studied to date induce apoptosis through the mitochondrial pathway, which is a conserved step for efficient viral replication and propagation through the host. However, the mechanisms by which this programmed cell death is induced varies drastically between virus genera, and even species. Previously, our workgroup reported that the LC from the FCV is located in the mitochondria, causing the relocation of Smac/DIABLO and the degradation of antiapoptotic proteins, such as survivin and XIAP, as well as triggering the mitochondrial apoptosis pathway, although the mechanism remains unknown [17]. Here, we aimed to determine if the LC from the FCV has the biochemical and functional characteristics of a viroporin, including the presence of a TMD, the presence of a one-sided helicoidal arrangement of basic amino acids, toxicity, a capacity to form homo-oligomers, and a capacity to permeate cellular membranes. Viroporins are small and often hydrophobic multifunctional viral proteins that locate and alter cellular membranes, forming protein pores or channels, facilitating viral replication in infected cells. Viroporins are involved in distinct aspects of viral replication, participating in endosome escaping, apoptosis, and calcium homeostasis alteration. In this regard, the only reported viroporin for the *Calciviridae* family was the recently characterized as an NS1-2 protein from the *Recovirus* genus, which impairs calcium homeostasis in cells [34]. Diverse viroporins from RNA viruses, such as the E protein from COVID-19 (CoV), p7 from the hepatitis C virus (HCV), and the 2B, 3A, and NS4A proteins from the poliovirus (PV) trigger apoptosis through the intrinsic pathway which is partially located in the mitochondria and it induces C cytochrome relocation to the cell cytoplasm [35], while other viroporins, such as US21 from the human cytomegalovirus (CMV) and 2B from the coxsackievirus (CaV) reduce cell susceptibility to apoptosis through a calcium homeostasis alteration [36,37]. Viroporins are classified into two broad groups, depending on the presence and orientation of one or two TMDs [38]. However, the recent discovery of viroporins that have more than two TMDs, such as the NS2A protein from dengue virus (DENV) [23], or that do not need a TMD or amphipathic helix for their function, such as the delta peptide from EBOV, suggest that other viral proteins with channel or pore activities could be classified into this group of proteins.

The delta peptide of EBOV is a secreted viroporin derived from the proteolytic cleavage of the viral glycoprotein (GP) that permeates membranes and induces cellular lysis through the presence of an intramolecular disulfide bond formed between two conserved cysteines, but not through its polybasic region or its amphipathic helix [18]. The membrane permeation mediated by the disulfide bonds is present in lytic proteins, such as defensins, which are antimicrobial peptides that share a molecular multidimensional signature known as γ-core, consisting of an intramolecular disulfide bond between two cysteine residues separated by 3 to 10 amino acids (GXCX_3-9_C) that folds in a loop conformation between two β-sheets. This motif is present in other proteins that interact with membranes, such as the prokaryotic chemokines and toxic peptides that have an evolutive span of over 2.6 billion years [39].

We found that the LC protein, produced from the proteolytic cleavage of the structural VP1 precursor protein [15], presents the defensins γ-core sequence GXCX_3-9_C in the CRI from several FCV strains and variants that have been analyzed. Moreover, the LC from other members of the genus *Vesivirus* also contain a γ-core sequence, and they lack the initial glycine residue, in concordance with what has been previously reported for the delta peptide from EBOV. The secondary structure prediction of the γ-core sequences shows two β-sheets flanking FCV WT-LC CRI, although the full LC protein tertiary structure prediction was unreliable (data not shown). This predicted a 3D arrangement of CRI both in its full protein form and as a peptide, showing that the LC has a similar antimicrobial peptide loop γ-core motif spatial localization as the defensins. Interestingly, this structure is not present in the mutant-LC-C40A protein that lacks the last cysteine residue in the CRI region. The apparent lack of a TMD and an amphipathic helix in the LC from the FCV is in accordance with what has been reported for some alpha defensins, where they do not require these domains to exert their anti-microbe effects. Having the LC protein tertiary structure might provide insights about its possible function as a defensin-like viroporin, similar to the delta peptide from EBOV.

The in vitro analysis of the WT-LC and mutant -LC-C40A proteins showed that both form disulfide bonds and oligomerize in non-reducing conditions. Moreover, the slight migration difference of the WT-LC and mutant-LC-C40A protein monomers in non-reduced conditions, in comparison with the reduced conditions, suggest that at least two intramolecular disulfide bonds are formed. These results correlate with the in silico predictions of DISULFIND server, which suggests that WT-LC protein from FCV forms three intramolecular disulfide bonds, similar to defensins [40]. However, no intramolecular disulfide bonds were predicted in the mutant LC-C40A protein. One possibility is that the DISULFIND server does not consider intermolecular disulfide bond formations. The fact that the mutant-LC-C40A protein oligomerizes in vitro suggests that inter-molecular disulfide bonds occur as a mechanism of oligomerization.

The higher oligomerization capability of the mutant LC-C40A can be explained by the loss of the 40-cysteine residue, which participates in an intramolecular disulfide bond formation, leaving an unpaired cysteine to form new intermolecular disulfide bonds that could lead to the increased oligomer formation and protein aggregation. Whether this conformation correlates with its reduced activity remains to be determined. 

Disulfide bond formation in eukaryotic cells is mostly limited to membrane-bound organelles, such as the ER and the mitochondrial periplasmic space, as the enzymes catalyzing its formation are located in these organelles and the cytoplasm maintains a reducing potential that makes the oxidation of thiol groups difficult. It has been well established that viral proteins use cell components in the ER to form disulfide bonds, as is the case with the hemagglutinin protein from the influenza virus [41] and the EBOV GP, which is a precursor of the previously discussed delta peptide [42]. However, a large DNA virus like the mimivirus, baculovirus, and the poxvirus encode viral proteins that are located in the cytoplasm and form disulfide bonds through the synthesis of their own sulfhydryl oxidases [43,44,45]. Alternatively, the formation of the intermolecular disulfide bonds on the µ protein of the reovirus occurs in the infected cell cytoplasm as a consequence of the redox imbalance of the cell through apoptosis induction, since these viruses do not encode for a disulfide isomerase or sulfhydryl oxidase [46]. This phenomenon has also been reported in cytoplasmic proteins of cell lines under oxidative stress [47]. As the sole expression of the LC protein from the FCV is sufficient to induce apoptosis [17], we infer that, if disulfide bonds are necessary for its function, their formation does not depend on other viral factors. 

The intrinsic toxicity of the LC protein from the FCV is another feature of viroporins, as its expression in bacteria impairs the formation of bacterial colonies [30]. However, we could not determine if the LC expression induces pores, since there were no changes in the OD_600_ of the LC-expressing *E. coli* (DE3)pLysS, a strain commonly used to assess membrane permeabilization by viroporins [23,30]. It is important to note that the T7 lysozyme expressed in the *E. coli* (DE3)pLysS strain has to relocate outside the cell to break down the bacterial cell walls and reduce OD_600_ levels. EBOV delta peptide forms pores that does not allow for the passage of molecules less than 10 kDa [18], and defensins such as the human neutrophil peptide 2 allows only a partial leakage of 4.4, but not of 18.9, kDa-sized particles [48]. The previous modelling of the EBOV delta peptide suggests that it forms chloride-selective channels [49], possiblly correlating with its reduced pore diameter. If the LC from the FCV has the ability to form channels or pores, needs to be further characterized.

TEM and immunogold labelling showed that the expression of WT-LC, but not the mutant-LC-C40A protein, induced the formation of structures resembling plasmolysis bays from bacteria exposed to mild and medium sucrose or glycerol hypertonic solutions [32,50,51], suggesting the induction of osmotic stress. The lack of plasmolysis bay formation by the expression of the mutant LC-C40A-expressing bacteria correlates with its reduced toxicity. Moreover, a lower amount of membrane-bound mutant-LC-C40A proteins and a different distribution of its inclusion bodies was also observed in comparison to the WT-LC-expressing cells. 

These differences in toxicity and apparent osmotic stress phenotype inductions may be related to the increased oligomer formation of the mutant LC-C40A protein, as its increased stability through disulfide bond formation can disrupt the membrane lysis function, similar to the VI protein from adenovirus (AdV) [52].

Previously, our research group reported that the mutant-LC-C40A was not capable of inducing the cytoplasmic localization of Smac/DIABLO and apoptosis [17]. Using TEM and immunogold assays, we found that both WT-LC and the mutant-LC-C40A are located at the mitochondria; however, while the mitochondria in Cyan-LC-expressing CrFK cells have the morphology of inner membrane herniation events in the MPT and apoptosis onset, the mutant LC-C40A failed to establish damaged mitochondria morphology correlating with its failure to induce osmotic stress in *E. coli*. These results suggest that apoptosis induced by the WT-LC protein from the FCV requires MPT, and cysteine 40 is important for FCV LC function but not for its subcellular localization. The MPT and internal mitochondrial membrane herniation is a reported event in apoptosis [33] through diverse causes, such as the mitochondrial permeability transition pore (PTP), long-lasting activation [53], or the actions of the proapoptotic Bax and Bak proteins [54]. To our knowledge, it is not known if this herniation is a cause or consequence of apoptosis onset. Thus, if the LC from the FCV cause MPT by inducing osmotic stress in the mitochondria (as seen in *E. coli* cells), or as a consequence of apoptosis induction through an unknown mechanism, similar to the relocation of Bax, as seen in the FCV replication cycle needs further investigation.

Taken together, the results presented here indicate that the LC from the FCV is a non-classical viroporin that mediates its activity through its disulfide bonds, similar to the delta peptide of EBOV.

## Data Availability

Not applicable.

## Supplementary Materials

The following supporting information can be downloaded at: https://www.mdpi.com/article/10.3390/v14030635/s1, Figure S1: Bioinformatical analysis of different defensins. Protein sequence of diverse defensins retrieved from the NCBI database and the protein sequence of the WT-LC protein were analyzed with TMHMM 2.0 and AmphipaSeek servers to predict TMDs or amphipathic helixes. Amino acid residues of predicted TMDs were plotted in red and the threshold for amphipathic helix (0 value) is indicated in the “y” axis. Table S1: Transmembrane domains and alpha helixes of diverse defensins.
