# Supplementary material for: The Feline Calicivirus Leader of the Capsid Protein Has the Functional Characteristics of a Viroporin
Source: Viruses. 2022 Mar 18;14(3):635. doi: 10.3390/v14030635 (PMC8955107; doi:10.3390/v14030635)
Supplement: Supplementary file 1 [file viruses-14-00635-s001.zip › viruses-1550453-supplementary.pdf]

viruses

MDPI

Supplementary Material

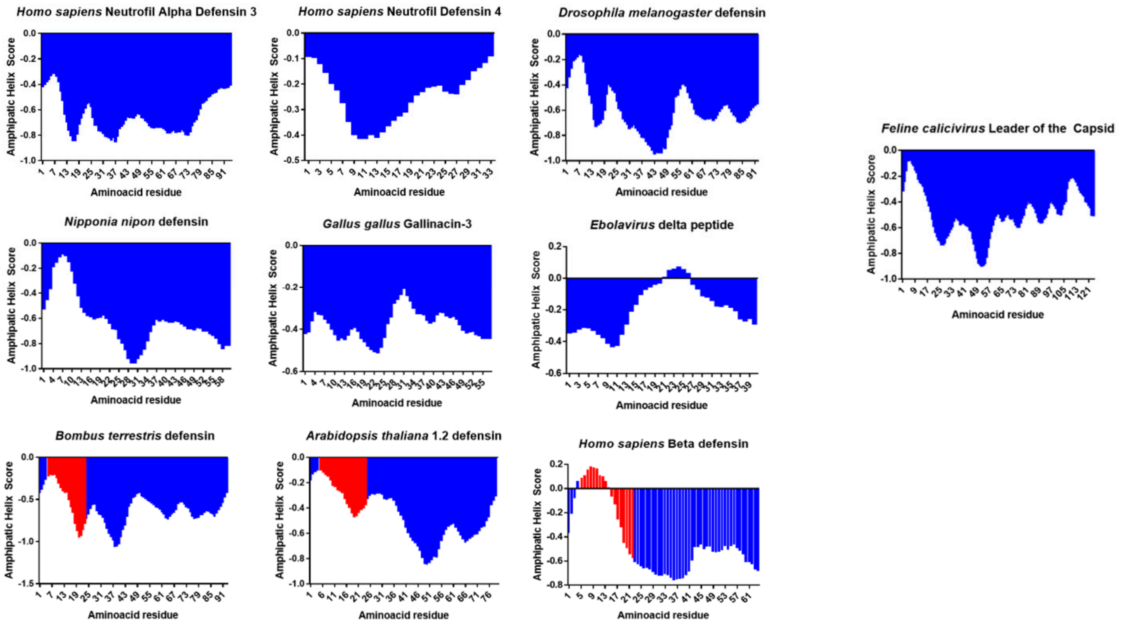

Figure S1. Bioinformatical analysis of different defensins. Protein sequence of diverse defensins retrieved from the NCBI database and the protein sequence of the WT-LC protein were analyzed with TMHMM 2.0 and AmphipaSeek servers to predict TMDs or amphipathic helixes. Amino acid residues of predicted TMDs were plotted in red and the threshold for amphipathic helix (0 value) is indicated in the "y" axis.

Table S1. Transmembrane domains and alpha helixes of diverse defensines.

Protein	Species	Transmembrane Domain Prediction	Amphipathic Helix Prediction
Defensin alpha 3 neutrophil specific	*Homo sapiens*	No	No
Neutrofil Defensin 4	*Homo sapiens*	No	No
Defensin	*Drosophila melanogaster*	No	No
Defensin	*Nipponia nipon*	No	No
Gallinacin 3	*Gallus gallus*	No	No
Delta peptide	*Ebolavirus*	No	Yes
Defenisn	*Bombus terrestris*	Yes	No
Defeinsin	*Arabidopsis thaliana*	Yes	No
Beta Defensin 106	*Homo sapiens*	Yes	Yes
Leader of the Capsid	*Feline calicivirus*	No	No
